# The association of exudation pattern with anatomical and functional outcomes in patients with Neovascular Age-Related Macular Degeneration

**Published:** 2019

**Authors:** Sibel Inan, Onur Polat, Mahmut Karadas, Umit Ubeyt Inan

**Affiliations:** * Afyonkarahisar Health Sciences University, Faculty of Medicine, Ophthalmology Department, Afyonkarahisar, Turkey; ** Afyonkarahisar State Hospital, Ophthalmology Clinic, Afyonkarahisar, Turkey; *** Dinar State Hospital, Ophthalmology Clinic, Afyonkarahisar, Turkey; **** Afyonkarahisar Parkhayat Hospital, Ophthalmology Clinic, Afyonkarahisar,Turkey

**Keywords:** ranibizumab, fluid configuration, age-related macular degeneration, subretinal fluid, cystoid fluid

## Abstract

**Objective.** To evaluate the correlation between visual outcomes and fluid configuration observed on spectral-domain optical coherence tomography (SD-OCT) in patients with wet age-related macular degeneration (AMD).

**Methods.** Sixty-five eyes of 53 patients with AMD who were administered intravitreal ranibizumab treatment with 12 months of follow-up were included in this retrospective study. Presence of intraretinal cystoid fluid (IRC) and pigment epithelial detachment (PED), thickness of subretinal fluid (SRF), central macular thickness (CMT), and central macular volume (CMV) were assessed.

**Results.** Subretinal fluid was observed in 29 eyes (45%), IRC in 36 eyes (55%), and PED in 39 eyes (60%). Baseline and final best-corrected visual acuity (BCVA) were 0.69±0.4 and 0.60±0.4 logMAR in the IRC negative group and 1.17±0.5 and 0.97±0.5 logMAR in the IRC positive group. BCVA was lower in IRC positive group (baseline p=0.001 and final=0.003); however, marked improvement was detected in both groups. Anatomic improvement and increased visual acuity were observed in groups with and without PED, IRC, and SRF. An inverse correlation was detected between pre-treatment CMT, IRC and post-treatment IRC, and final BCVA.

**Conclusion.** Significant visual and anatomic improvement was observed after one-year of ranibizumab treatment regardless of fluid configuration. However, the presence of IRC was observed to be associated with worse visual acuity. Baseline retinal fluid configuration may have prognostic effects on functional success in patients treated with ranibizumab for wet AMD.

**Abbreviations.** AMD = Age-related macular degeneration, VEGF = Vascular endothelial growth factor, IRC = intraretinal cystoid fluid, PED = pigment epithelial detachment, SRF = subretinal fluid, SD-OCT = spectral-domain ocular coherence tomography, IVR = intravitreal ranibizumab, BCVA = best-corrected visual acuity, FFA = fundus fluorescein angiography, CMT = central macular thickness, CMV = central macular volume

## Introduction

Age-related macular degeneration (AMD) is the leading cause of permanent vision loss in individuals ≥ 65 years of age in developed countries [**1**]. Vascular endothelial growth factor (VEGF), which plays a significant role in angiogenesis and vascular permeability, is the most important molecule held responsible for the pathogenesis [**2**].

Use of anti-VEGF agents has an essential role in the current treatment of AMD [**3**]. Favorable visual outcomes were obtained in controlled-randomized clinical trials. However, considering that monthly ranibizumab injection is not feasible in real-life practice, individualized treatment regimens such as pro re nata (PRN) and “treat and extend” have been developed with successful visual outcomes [**4**-**8**]. In individualized treatments, the decision on repeated injections is based on visual acuity and central retinal thickness changes [**9**]. 

Various retinal morphologic markers such as intraretinal cystoid fluid (IRC), pigment epithelial detachment (PED), thickness of subretinal fluid (SRF), posterior vitreous detachment, vitreomacular adhesion and vitreomacular traction were shown to be important to assess the retina with spectral-domain optical coherence tomography (SD-OCT) [**10**]. Particularly, localization of the fluid compartment leading to impairment of macular morphology during the onset and course of the disease was reported to be a predictor of response to treatment and also a guide in individualized treatments [**10**,**11**]. Thus, SD-OCT may present visual biomarkers to assess the prognosis of anti-VEGF treatment [**10**,**11**].

While various morphologic retinal changes, which may have a prognostic effect on the final visual acuity in AMD patients, have been investigated in the literature, investigations are still ongoing in a controversial manner [**12**-**14**]. The present study was performed to investigate the correlation between the baseline retinal fluid configuration and the visual outcomes at month-12 in wet-AMD patients receiving intravitreal ranibizumab treatment.

## Methods

Files of patients who were diagnosed with wet-AMD treated with intravitreal ranibizumab (IVR) injection and followed for at least 12 months were retrospectively evaluated. The study was designed in accordance with the principles of the Helsinki Declaration, and approval was obtained from the ethical committee. Patients were informed regarding the study, and informed consent was obtained from each patient.

Inclusion criteria were older than 50 years of age, diagnosed with naïve wet-AMD, treated with IVR injection, followed for at least 12 months and a baseline best-corrected visual acuity (BCVA) from 20/ 200 to 20/ 25. Patients with intravitreal or intracameral anti-VEGF injections other than ranibizumab, vitreomacular interface diseases, RPE discontinuity, baseline BCVA worse than 20/ 200, previous vitreoretinal surgery, retinal pathology other than wet-AMD, inflammatory eye disease, and glaucoma were excluded from the study. 

At pre-treatment examination, all patients underwent BCVA assessment with ETDRS chart, anterior segment examination with slit-lamp, intraocular pressure measurement, and detailed post-dilation fundus examination. Before treatment, fundus fluorescein angiography (FFA) and SD-OCT (Cirrus HD OCT, Carl Zeiss Meditec, Dublin, CA, USA) imaging were performed. During the control visits, each patient underwent ophthalmologic examination and SD-OCT imaging while FFA was performed only if deemed necessary. Treatment involved three consecutive monthly IVR injection followed by additional injections administered as needed based on the OCT and visual acuity findings.

Patient demographics and ocular features were recorded. According to BCVA results and fluid configuration observed on SD-OCT, the presence of IRC, presence and height of PED, CRT, central macular thickness (CMT) and central macular volume (CMV) measurements were recorded before treatment and at 12 months. Upon observing measurement errors due to inaccurate segmentation drawing with the device, measurements were obtained manually following correction. The results obtained with SD-OCT and BCVA were compared by means of subgroup analysis assessment.

Statistics software SPSS 18.0 (SPSS, Inc., IL, USA) was used for the statistical analysis. Differences between the pre-treatment and month 12 values of variables were compared using the paired t-test. Independent samples t-test test was employed to assess inter-group differences. Pearson’s correlation analysis was used for the correlation analysis. Assessments were performed at 95% confidence interval, and p<0.05 was considered statistically significant.

## Results

The study included 65 eyes of 53 patients with wet-AMD. There were 26 females and 27 males. The mean age was 68.3±9.1 years. The mean number of IVR injection was 4.76±0.82. SRF was observed in 29 eyes (45%), IRC in 36 eyes (55%), and all-size PED in 39 eyes (60%). While pre-treatment BCVA was 0.97±0.54 logMAR, it was improved to 0.81±0.53 logMAR at 12 months with a statistically significant difference (p=0.001). Baseline CMT, CMV, SRF thickness and PED height were 367.5±104μm, 11.0±1.67mm3, 212.3±125.4μm, and 287.0±188.0μm, respectively while the final CMT, CMV, SRF thickness and PED height were 283.9±107μm, 9.8±1.2mm3, 193.3±122.2μm, and 211.5±130.2μm, respectively (p=0.001, p=0.001, p=0,43, and p=0.001, respectively).

While 29 patients (45%) had SRF before treatment, this number was 9 (14%) at 12 months after treatment. BCVA, CMT, and CMV values detected in eyes with and without subretinal fluid before and after treatment are summarized in **Table 1**. Accordingly, CMT and CMV were significantly low in the group with no SRF before treatment. No significant difference was detected between the two groups with respect to BCVA, CMT and CMV measurements after treatment. 

**Table 1:** BCVA and OCT measurements in subretinal fluid groups

	SRF (-)	SRF(+)	p value
BCVA- month 0	0.95±0.53	1.02±0.55	0.66
BCVA- month 12	0.77±0.52	0.87±0.54	0.43
CMT- month 0	352.8±113.1	388.5±91.7	0.03
CMK- month 12	270.3±83.5	300.6±132.8	0.58
CMV- month 0	10.3±1.5	11.8±1.5	<0.001
CMV- month 12	9.6±0.9	10.1±1.3	0.20
SRF = Subretinal fluid, OCT = Optic coherence tomography, BCVA = Best-corrected visual acuity, CMK = Central macular thickness, CMV = Central macular volume			

Intraretinal cystoid fluid was present in 36 patients (55%) before treatment. At 12 months of treatment, this number was 20 (31%). BCVA, CMT, and CMV values detected before and after treatment by the presence of IRC are presented in **Table 2**. Accordingly, BCVA was significantly more favorable, and CMT and CMV were low in the group with no IRC at baseline. After 12 months of treatment, a significant difference was observed between the groups in terms of BCVA measurements. The IRC (+) group had statistically lower pre-treatment and post-treatment BCVA values compared to the IRC (-) group. 

**Table 2:** BCVA and OCT measurements in intraretinal cystoid fluid groups

	IRC (-)	IRC (+)	p value
BCVA- month 0	0.69±0.4	1.17±0.5	<0.001
BCVA- month 12	0.60±0.4	0.97±0.5	0.01
CMT- month 0	313.4±47.9	408.5±116.9	<0.001
CMK- month 12	268.8±48.5	295.4±136.5	0.84
CMV- month 0	10.5±1.2	11.4±1.8	0.028
CMV- month 12	9.6±0.6	9.9±1.4	0.62
IRC = Intraretinal cystoid fluid, OCT = Optic coherence tomography, BCVA = Best-corrected visual acuity, CMK = Central macular thickness, CMV = Central macular volume			

While 40 patients (61.5%) had PED before treatment, this number decreased to 33 (50.8%) after 12 months of treatment. Pre- and post-treatment BCVA, CMT, and CMV detected in the eyes with and without PED are summarized in **Table 3**. Accordingly, no statistically significant difference was seen between the groups in terms of the values obtained before and after 12 months of treatment. 

**Table 3:** BCVA and OCT measurements in pigment epithelial detachment groups

	PED (-)	PED (+)	p value
BCVA- month 0	1.01±0.5	0.95±0.5	0.65
BCVA- month 12	0.81±0.5	0.81±0.5	0.99
CMT- month 0	369.5±91.2	366.3±113.1	0.91
CMK- month 12	284.1±109.1	284.1±109.1	0.89
CMV- month 0	11.2±1.6	10.8±1.7	0.42
CMV- month 12	9.7±1.1	9.8±1.1	0.87
PED = Pigment epithelial detachment, OCT = Optic coherence tomography, BCVA = Best-corrected visual acuity, CMK = Central macular thickness, CMV = Central macular volume			

When the cases with SRF, IRC and/ or PED in the same eye were evaluated, post-treatment BCVA was observed to be better than pre-treatment in all groups; however, the difference was not statistically significant (**Table 4**). Mean pre-treatment BCVA was the highest in PED(-)/ IRC(-) patients. The lowest mean BCVA was observed in SRF(+)/ IRC(+) patients. Similarly, mean highest post-treatment BCVA was seen in PED(-)/ IRC(-) patients, and the lowest BCVA was observed in BCVA SRF(+)/ IRC(+) patients.

**Table 4:** BCVA and OCT measurements in cases with SRF, IRC, and/ or PED

	BCVA(month 0)	BCVA (month 12)	p value
SRF (-)/IRC(-)	0.69±0.4	0.57±0.5	0.65
SRF (+)/IRC (+)	1.32±0.5	1.09±0.6	0.94
SRF (-)/PED (-)	1.03±0.5	0.83±0.5	0.91
SRF (+)/PED (+)	1.05±0.5	0.93±0.4	0.89
PED (-)/IRC (-)	0.63±0.4	0.48±0.4	0.42
PED (+)/IRC (+)	1.19±0.5	0.98±0.5	0.87
SRF = Subretinal fluid, IRC = Intraretinal cystoid fluid, PED = Pigment epithelial detachment, OCT = Optic coherence tomography, BCVA = Best-corrected visual acuity, CMK = Central macular thickness, CMV = Central macular volume			

Correlation analysis revealed a significant correlation between pre-treatment BCVA and pre-treatment CMT, CMV, SRF thickness and the presence of IRC (p<0.001, r=0.47, p=0.005, r=0.34, p=0.048, r=0.32, p<0.001, r=0.46, respectively). No significant correlation was detected with the presence or height of PED. There was a significant correlation between post-treatment BCVA and pre-treatment CMT, pre-treatment IRC and post-treatment IRC (p=0.004, r=0.35, p=0.004, r=0.35, p=0.001, r=0.401).

## Discussion

In our study, we detected an inverse correlation between CMT and IRC, and the final visual acuity achieved at month-12 in wet-AMD patients treated with PRN protocol following three consecutive doses of intravitreal ranibizumab injection. While the presence of SRF and PED had unfavorable effects on the baseline and final visual acuity, the effect was not statistically significant.

Detailed analysis of retinal and subretinal compartments such as SRF, IRC and PED with SD-OCT was reported to define the characteristic impact of treatment [**15**,**16**]. As it is known, depending on the amount of VEGF increase in wet-AMD pathogenesis, leakage occurs from neovascular veins and IRC develops through the anatomic and functional impairment in intraretinal structures. Studies have revealed that IRC is the morphologic parameter that provides the most rapid response to intravitreal anti-VEGF treatment, resulting in improvement in retinal microstructure and neurosensorial retina and also most rapidly leading to recurrence upon interruption of treatment. Therefore, IRC has been reported to be the most sensitive morphologic parameter [**10**,**16**]. While no association was seen between the presence of subretinal fluid after a loading dose of 4 injections in a study, intraretinal cystic fluid was associated with worse vision. However, VA at six months, structural outcomes, and the number of injections were not different between the groups. This finding, along with the presence of intraretinal fluid at baseline leading to delay in response to treatment, indicates that six-month treatment results are similar [**11**]. In our study, we observed that the presence of pre-treatment IRC had an unfavorable effect on the final visual acuity, which was in line with the literature. Accordingly, the final visual acuity achieved by patients with pre-treatment IRC was less favorable compared to the group of patients without pre-treatment IRC. This may be explained by the unfavorable changes or injury induced by pre-treatment IRC in intraretinal structures, although the rapid response to treatment is achieved [**17**,**18**]. 

In literature, answers to the question of why patients with SRF and PVD achieve improvement with less treatment is still being investigated. In contrast with ICR, the response to intravitreal anti-VEGF treatment is relatively slow with SRF and recurrence occurs after a longer period upon the interruption of treatment. The reason for this is the fact that access of the intravitreal drug into the subretinal region is more difficult, and liquid absorption also takes a longer time [**17**,**18**]. A study demonstrated subretinal fluid accumulation as the marker best indicating disease recurrence and the efficacy of anti-VEGF treatment [**19**]. Subretinal fluid volume may be a predictive parameter for the initial functional response in anti-VEGF treatment strategy. In addition, the optic density rate of the fluid may reflect the situation of the blood-retina barrier and may be used for the pathophysiological differentiation and prognostic purposes [**20**]. In clinical trials, SRF was found to be associated with more favorable VA [**21**,**22**]. However, no clear cause explaining the association between SRF and better VA was defined [**23**]. This result in line with our study results may be related to the absence of a statistically negative impact of pre-treatment SRF on the final visual acuity achieved after treatment, the lesser injury induced by intraretinal microstructure, and thus the slower recurrence following functional and anatomic improvement.

There are a limited number of reports regarding the effects of pre-treatment PED on final visual acuity and visual prognosis in the literature. Similar to IRC, it is reported to be associated with an aggressive AMD subtype and may require intensive treatment [**10**,**16**]. In addition, similar to SRF and with a similar mechanism, it is reported that treatment response occurs late in patients with PED and that a negative predictive effect may develop on visual prognosis when IRC and SRF co-exist [**10**,**16**]. In patients with serous and vascular PED, ranibizumab was reported to be a safe and effective treatment modality, and the anatomic response in PED did not show a direct correlation with the visual response [**24**]. In another study, while the dome-shaped PED was associated with a lower central macular thickness, it did not affect the response to treatment. While the existence of intraretinal cystic fluid was related to the worse vision during the follow-up in CATT and other large-scale studies, the presence of sub-RPE fluid was found to have a relatively smaller effect on vision [**6**,**11**,**23**]. In our study, we observed that the presence of pre-treatment PED did not induce any unfavorable effects on the final visual acuity. Similar to SRF, this may be related to the lesser injury induced in the intraretinal microstructure. However, in clinical practice, one should consider that retinal fluid patterns and morphologic structures might co-exist in AMD patients. In this case, visual and functional outcomes may vary depending on the concomitant fluid pattern.

The limitations of our study include the retrospective design and small sample size. Other factors, including genetic and demographic factors, could be considered. 

In conclusion, IRC was observed to be the fluid configuration with the most unfavorable effect on visual outcomes. Retinal fluid configurations may have different effects on treatment success. Upon determining the effects of morphologic retinal structures on anatomic and functional success, they may be used as prognostic factors and effectively guide the way in setting alternative treatment modalities. 

**Disclosure Statement**

The authors declared no conflict of interest and that this study received no financial support.
